# Knee laxity modifications after ACL rupture and surgical intra- and extra-articular reconstructions: intra-operative measures in reconstructed and healthy knees

**DOI:** 10.1007/s00167-015-3653-1

**Published:** 2015-06-03

**Authors:** Pierre Imbert, Claudio Belvedere, Alberto Leardini

**Affiliations:** 1 Department of Knee Surgery, Clinique Notre Dame De La Merci, 215, Avenue du Maréchal Lyautey, 83700 Saint Raphaël, France; 2 Movement Analysis Laboratory, Istituto Ortopedico Rizzoli, Bologna, Italy

**Keywords:** Knee kinematics, Laxity measurements, ACL reconstruction, Contralateral healthy knee, Knee compartments, Extra-articular procedure, Anterolateral reinforcement, Anterolateral ligament

## Abstract

**Purpose:**

Quantifying the effects of anterior cruciate ligament (ACL) deficiency on knee joint laxity is fundamental for understanding the outcomes of its reconstruction techniques. The general aim of this study was to determine intra-operatively the main modifications in knee laxity before and after standard isolated intra-articular and additional extra-articular anterolateral reinforcement. Our main hypothesis was that laxity abnormalities, particularly axial rotation, can still result from these ACL reconstruction techniques.

**Methods:**

Thirty-two patients with primary ACL deficiency were analysed by a navigation system immediately before and after each of the two reconstructions. Laxity measurements in terms of knee translations and rotations were taken during the anteroposterior drawer test, with internal–external rotation at 20° and 90° of flexion, and varus–valgus and pivot-shift tests. All these laxity measures were also taken originally from the contralateral healthy knee.

**Results:**

With respect to the contralateral healthy knee, in the ACL-deficient knee significantly increased laxity (expressed in %) was found in the medial compared with that of the lateral compartment, respectively, 115 and 68 % in the drawer test at 20° flexion, and 55 and 46 % at 90° flexion. In the medial compartment, a significant 35 % increment was also observed for the coupled tibial anteroposterior translation during axial knee rotation at 20° of flexion. After isolated intra-articular reconstruction, normal values of anteroposterior laxity were found restored in the pivot-shift and drawer tests in the lateral compartment, but not fully in the medial compartment. After the reinforcement, laxity in the medial compartment was also found restored in the axial rotation test at 20° flexion.

**Conclusion:**

In ACL reconstruction, with respect to the contralateral knee, intra-articular plus additional anterolateral reinforcement procedures do not restore normal joint laxity. This combined procedure over-constrained the lateral compartment, while excessive laxity still persists at the medial one.

**Level of evidence:**

III.

## Introduction

Over the last 30 years, advancements in anterior cruciate ligament (ACL) reconstruction techniques have enabled less invasive knee surgery and a more rapid recovery for the patients. However, the occurrence of secondary degenerative changes despite successful knee joint stabilization [45, 58] and excessive residual internal rotation [1, 17] have led to the development of surgical reconstructions intended to provide better long-term rotational knee mobility and stability. Although there are still contradictory results concerning the performance of the very demanding double-bundle ACL reconstruction technique [56, 61], the extra-articular anterolateral reinforcement has recently attracted renewed interest, partially because of recent findings on the anatomy of the anterolateral ligament [11]. However, long-term follow-up studies of intra- and additional extra-articular reconstruction have not shown improvement in reducing these degenerative changes [49, 54, 62].

Modern measurement systems, such as electromagnetic or inertial motion units or digital image-based motion analysis, claim to provide in vivo knee motion also for joint laxity evaluation, but these non-invasive systems have not yet been fully validated for clinical applications [3, 48]. To date, only invasive quantitative measurement systems using bony trackers, such as surgical navigation systems [12, 30] or radiostereometry with tantalum beads [25], have produced reliable bone motion data [47]. Although surgical navigation systems are able to perform reliable measurements of joint laxity at the ACL-injured knee [23, 48], the corresponding original pre-injury data are obviously unknown. Moreover, there are no data about normal joint laxity derived from bone tracking in vivo in healthy knees.

For these reasons, laxity measurements were taken by the present authors using a surgical navigation system, in the ACL-deficient knee (ADK) before reconstruction, but also, very originally, at the contralateral healthy knee (CHK) [23]. The aim of the present study was to determine intra-operatively the main modifications in knee laxity before and after these two surgical reconstructions, to evaluate the effectiveness of these two procedures immediately after surgery, for the first time having the contralateral as the reference. Our main hypothesis was that the intra-articular followed by the extra-articular ACL reconstruction technique may still result in laxity abnormalities at the knee joint, in terms of both residual laxity and over-constrained joint conditions. In particular, whereas the limiting effects in axial rotation of the additional ALR have been pointed out [42, 52], the separate residual laxity at the two knee compartments is still an important topic in the clinical debate on the surgical choices for ACL reconstruction. The present results therefore should contribute to the understanding and large debate of intra- and extra-articular ACL reconstruction techniques [9, 11, 14, 31].

## Materials and methods

To address this thoroughly, knee joint rotation and translation measurements were taken intra-operatively by an accurate surgical navigation system while performing a number of manual laxity tests, before (the ADK) and after each of these two reconstruction techniques (IAR and ALR). To remove inter-subject variability, laxity measures were also taken originally from the contralateral limb (the CHK).

Thirty-two ACL reconstructions by a single experienced surgeon were analysed in as many patients within one and half years of the injury. The inclusion criteria were as follows: (a) isolated ACL rupture, detected by no varus–valgus laxity, meniscal lesion or cartilage damage as shown by a questionnaire, physical examination and MRI and (b) uninjured contralateral knee, as assessed by a questionnaire and physical examination. Pre-operative clinical assessment was performed by using the International Knee Documentation Committee scoring system [19]. In the ADK, all patients had joint instability (score C or D), with no clinical or radiological evidence of any other ligamentous lesion, degenerative change or meniscus lesion. In all patients, the CHK was stable with no major ligament injuries or degenerative changes (score A or B). All patients were asked before surgery to allow intra-operative data collection from both the ADK and CHK, according to an established technique [41] suitably adapted.

### The navigation system and the measurements

During surgery, knee joint motion measurements were taken according to a technique already described in detail [23], by using an image-free passive-optical surgical navigation system (Praxim Medivision, La Tronche, France; tested accuracy and resolution of 1° and 1 mm [12]). This system, however, was used only for the intra-operative laxity measurements, not to guide the ACL reconstruction. The system provides in real time knee flexion–extension, varus–valgus and internal–external rotation, i.e. the axial rotation, by means of bone trackers implanted in the femur and tibia [12, 23]. A third pointer-like cluster was used to digitize percutaneously the following anatomical landmarks to be used to define relevant reference frames at the femur and tibia: the medial and lateral epicondyles, the medial and lateral malleoli, the most prominent part of the tibial tuberosity, and the most medial and lateral ridge of the tibial plateau [23]. The anteroposterior laxity was defined as the range, expressed in millimetres, of anteroposterior translation in the tibial transverse plane of a point of the femur: this was the lateral or medial compartment, or the central aspect, according to the test analysed.

### The experimental tests and the joint conditions

These knee joint rotations and translations were collected during a number of manual laxity tests [20, 28] (Table 1). The varus–valgus laxity test was performed in full extension rather than at 20° of flexion to avoid the automatic internal tibial rotation and knee valgisation by the anterior gliding of the femoral condyle on the tibial plateau [43]. The pivot-shift test [34] was a reduction-type manoeuvre, utilizing high valgus stress with a slight internal tibial rotation; in external rotation, posteromedial lesions could have been revealed [16], but this was not relevant for the present study.

**Table 1:** Manual knee laxity tests performed during surgery, and relevant joint motion variables analysed, as measured by the navigation system (integer values)

Manual laxity test	Relevant knee joint measurements, range of
Anteroposterior drawer test at 20° of flexion	Cnteroposterior femoral translation (mm), distinguished between the medial and the lateral compartments
Anteroposterior drawer test at 90° of flexion, with the foot flat on the table in neutral rotation	Anteroposterior femoral translation (mm), distinguished between the medial and the lateral compartments
Internal–external rotation at 20° of flexion	Axial rotation (°), and anteroposterior femoral translation (mm) distinguished between the medial and the lateral compartments
Internal–external rotation at 90° of flexion	Axial rotation (°), and anteroposterior femoral translation (mm) distinguished between the medial and the lateral compartments
Varus–valgus laxity test at 0° of flexion	Joint rotation in the frontal plane (°)
Pivot-shift test	Axial rotation (°), and anteroposterior femoral translation (mm) of the central point

### The surgical techniques

**Fig. 1:**
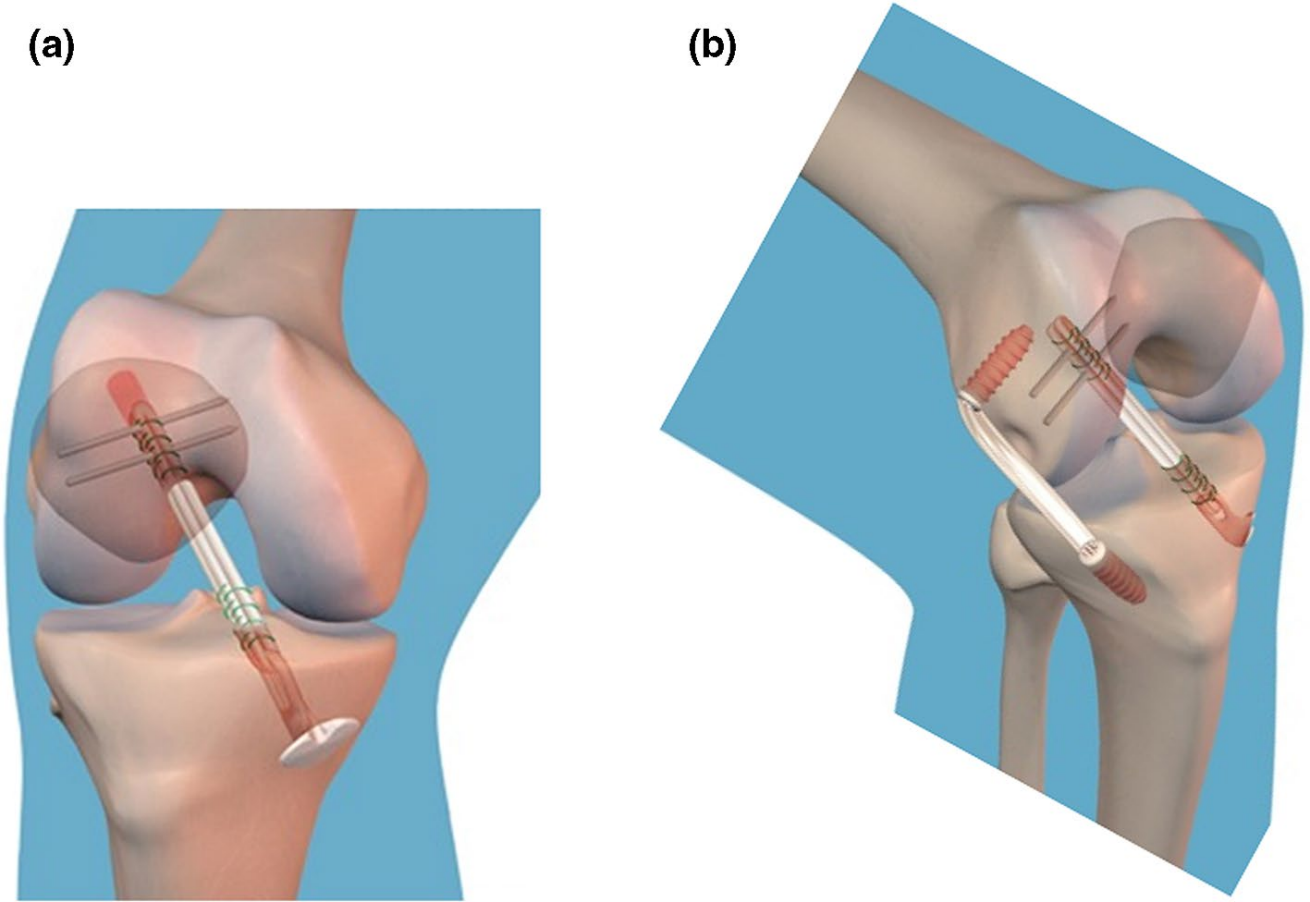
Diagrams of the two surgical procedures: **a** isolated intra-articular reconstruction (IAR) with autologous semitendinosus in a four-bundle montage; **b** intra-articular reconstruction with the extra-articular anterolateral reinforcement (ALR) using autologous gracilis tendon in a two-bundle montage

These measurements were taken in the CHK first, fixing the clusters by Steinmann pins. The clusters were then moved to the ADK and fixed by minimally invasive unicortical Schanz screws. The manual laxity tests were repeated and collected by the system before surgery, after isolated IAR and finally after the additional minimally invasive ALR. For IAR, the autologous semitendinosus tendon was taken and prepared for a short four-bundle single-tunnel montage, whose extremity graft was fixed 15 mm inside the tibial bone tunnel. The femoral tunnel was drilled by the anteromedial portal, and femoral fixation was secured with a transverse cross-pin system inserted close to the femoral entry tunnel (Fig. 1a). The graft was pre-tensioned for 10 min at 10 Newton and subsequently fixed at 30° of knee flexion with manual tensioning enabling the knee to be lifted from the operating table. The ALR [24] involved folding the autologous gracilis tendon and making a free 11-cm-long graft, inserted from a femoral position 1 cm proximal and posterior to the lateral epicondyle to a tibial margin position against the posterior aspect of the Gerdy’s tubercle. Interference screws inside bone tunnels were used for femoral and tibial fixation of the graft. Drilling the tunnels, inserting the graft and fixing it with interference screws were performed through two 1.5-cm-long incisions. Between them, graft application was completed by blunt dissection under the fascia lata through the distal incision with crocodile forceps (Fig. 1b). The graft was secured with 90° of knee flexion and 0° of internal–external rotation.

Written informed consent was obtained by each patient, under a relevant approval of the Ethics Committee of the “Notre Dame de la Merci” hospital (Project “Quantitative assessment of joint laxity by a navigation measurement system”, dated 15 September 2007).

### Statistical analysis

The patient population size analysed in this study is based on preliminary data [23]. Particularly, these 32 patients meet the criteria for achieving differences between measurements where the level of magnitude of the standard deviation is equal or less than that of the corresponding mean value for each analysed variable, with 80 % statistical power and an α-level of 0.05. The results in terms of rotations and translations were reported in standard box plots for the four conditions: CHK, and the three on the surgically treated knee (ADK, IAR and IAR + ALR). For all these motion variables and over the six laxity tests analysed, correlations between values were sought. The Pearson product–moment correlation coefficient (*R*) and its squared form, the coefficient of determination (*R* ^2^), were used to determine the relationships between the four conditions, over all tests and knee compartments. Statistical significance was taken for *p* values smaller than 0.05. All statistical analyses were made with the MATLAB^®^ software package (the MathWorks Inc., Natick, MA, USA).

## Results

No surgical complications occurred, and no persistent pain was reported by the patients.

**Fig. 2:**
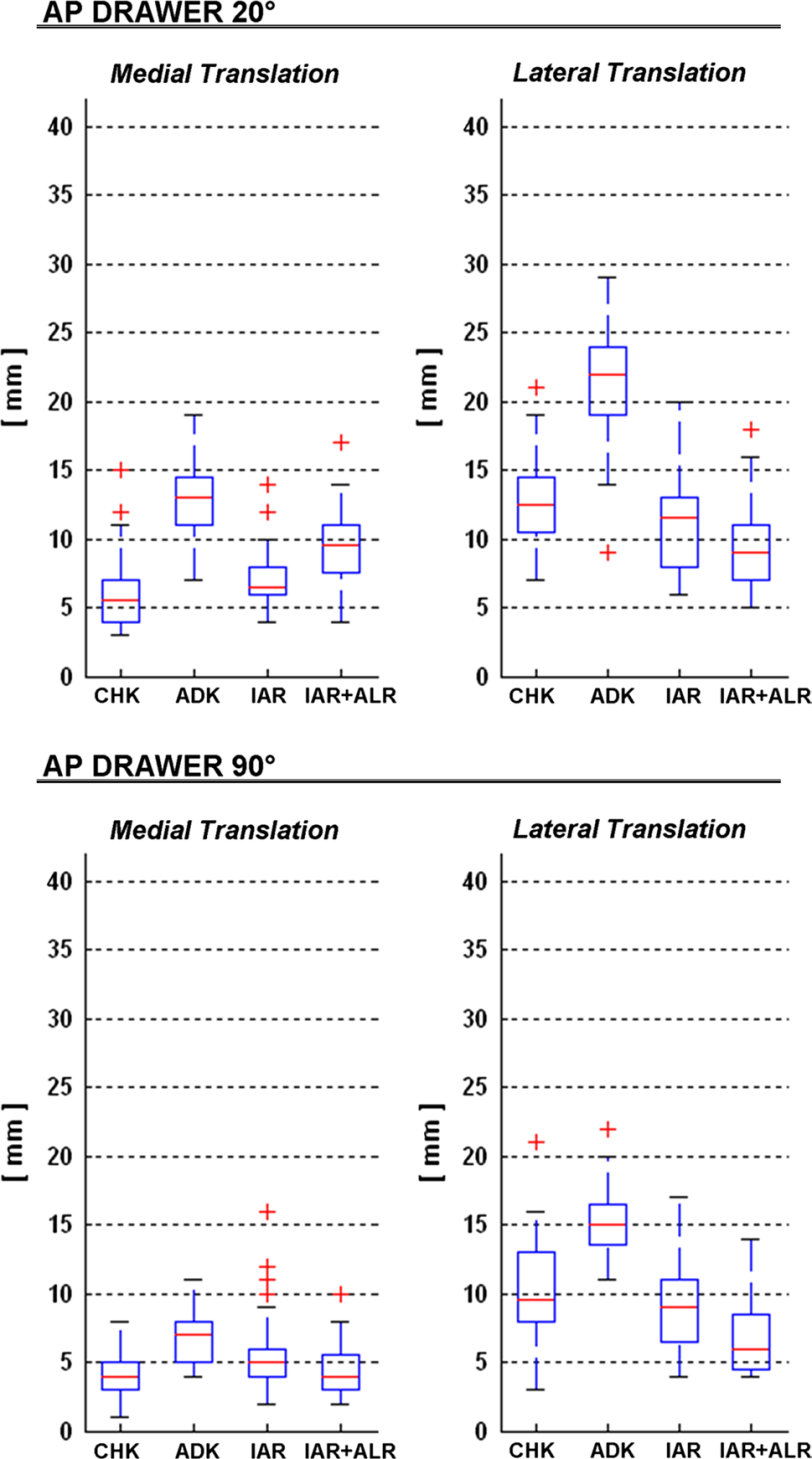
*Box plots* of the anteroposterior translations in the medial (*left*) and the lateral (*right*) compartments, during the anteroposterior drawer test at 20° (*top*) and 90° (*bottom*) of knee flexion, at each of the four knee conditions (*CHK* contralateral healthy knee, *ADK* ACL-deficient knee, *IAR* intra-articular reconstruction, *IAR* + *ALR* intra-articular reconstruction + anterolateral reinforcement). In each plot, the *boxes* have *lines* at the *lower*, *median* and *upper* quartile values over the whole patient cohort; the *whisker lines* extending from each end of the *box* show the extent of the rest of the data; values for any outliers are reported with a *red cross* beyond the ends of the whiskers

During the anteroposterior drawer test, in CHK the anteroposterior laxity was higher at 20° than at 90° (*p* < 0.00), and in every single knee that in the lateral compartment was more than twice the value of that in the medial compartment (Fig. 2; Table 2). In ADK, laxity was significantly larger; the percentage difference of the mean values was higher in the medial (115 % at 20° and 55 % at 90°) than in the lateral compartment (68 % at 20° and 46 % at 90°). In general, laxity was obviously much smaller after IAR and additional ALR than at ADK, though the values observed at the CHK were not fully restored. Particularly, at 20° flexion, laxity in the medial compartment at CHK (about 6 mm) was somehow restored with IAR (about 7, n.s.), but this result deteriorated after IAR + ALR (about 9, *p* < 0.00); in the lateral compartment, CHK laxity (13 mm) was already over-constrained with IAR (11 mm, *p* = 0.025) and even more with IAR + ALR (9 mm, *p* < 0.00). At 90° flexion, laxity in the medial compartment at CHK was not restored fully with IAR (*p* = 0.046), but it was somehow restored with IAR + ALR (n.s.); in the lateral compartment, CHK laxity was restored with IAR (n.s.), but over-constrained with IAR + ALR (*p* < 0.00).

**Table 2:** Knee motion variables during the manual laxity tests: mean ± standard deviations values at each of the four conditions (CHK, ADK, IAR, IAR + ALR) and relevant statistical-based comparisons, in terms of *p* values, and as Pearson product–moment correlation coefficient of determination (*R* ^2^)

	Drawer test at 20° translations (mm)		Drawer test at 90° translations (mm)		Internal–external rotation test at 20°			Internal–external rotation test at 90°			Varus–valgus test	Pivot-shift test	
	Medial	Lateral	Medial	Lateral	Axial rotation (°)	Medial (mm)	Lateral (mm)	(°)	Medial (mm)	Lateral (mm)	(°)	(°)	Central (mm)
CHK	6.0 ± 2.9	12.8 ± 3.3	4.3 ± 1.6	10.3 ± 3.5	23.9 ± 5.8	16.5 ± 5.5	19.2 ± 4.1	37.5 ± 6.5	22.9 ± 5.6	30.3 ± 4.4	3.0 ± 1.2	16.3 ± 3.6	8.8 ± 3.3
ADK	12.8 ± 2.9	21.5 ± 4.3	6.7 ± 1.7	15.1 ± 2.5	26.4 ± 5.5	22.3 ± 5.2	20.4 ± 4.6	39.0 ± 6.1	26.4 ± 3.9	32.6 ± 3.7	4.4 ± 1.6	21.5 ± 4.5	14.9 ± 5.5
IAR	7.1 ± 2.2	10.9 ± 3.4	5.5 ± 3.0	8.9 ± 3.1	23.8 ± 5.7	18.0 ± 4.6	19.5 ± 4.1	33.6 ± 5.7	23.8 ± 5.6	28.4 ± 4.3	4.0 ± 1.2	18.1 ± 4.6	9.1 ± 4.8
IAR + ALR	9.3 ± 3.0	9.5 ± 3.0	4.5 ± 1.7	6.6 ± 2.5	20.1 ± 4.5	17.1 ± 3.5	14.9 ± 4.2	23.7 ± 5.7	17.9 ± 4.6	19.3 ± 4.6	3.6 ± 1.1	15.3 ± 4.8	8.8 ± 5.3
*P*_CHK → ADK_	0.00	0.00	0.00	0.00	0.07	0.00	0.24	0.34	0.005	0.024	0.00	0.00	0.00
*P*_CHK → IAR_	0.08	0.025	0.046	0.09	0.98	0.26	0.74	0.012	0.55	0.09	0.001	0.09	0.76
*P*_CHK → IAR + ALR_	0.00	0.00	0.59	0.00	0.005	0.61	0.00	0.00	0.00	0.00	0.039	0.35	0.95
*P*_ADK → IAR_	0.00	0.00	0.06	0.00	0.07	0.001	0.4	0.00	0.032	0.00	0.28	0.004	0.00
*P*_ADK → IAR + ALR_	0.00	0.00	0.00	0.00	0.00	0.00	0.00	0.00	0.00	0.00	0.019	0.00	0.00
*P*_IAR → IAR + ALR_	0.001	0.09	0.11	0.002	0.005	0.41	0.00	0.00	0.00	0.00	0.12	0.022	0.84
$R_{{{\text{CHK}} \to {\text{ADK}}}}^{2}$	0.59	0.58	0.36	0.38	0.05	0.23	0.02	0.01	0.12	0.08	0.21	0.30	0.32
$R_{{{\text{CHK}} \to {\text{IAR}}}}^{2}$	0.05	0.08	0.06	0.05	0.00	0.02	0.00	0.10	0.01	0.05	0.17	0.05	0.00
$R_{{{\text{CHK}} \to {\text{IAR + ALR}}}}^{2}$	0.26	0.22	0.00	0.28	0.12	0.00	0.21	0.57	0.20	0.61	0.07	0.01	0.00
$R_{{{\text{ADK}} \to {\text{IAR}}}}^{2}$	0.55	0.67	0.05	0.55	0.05	0.16	0.01	0.18	0.07	0.22	0.02	0.13	0.25
$R_{{{\text{ADK}} \to {\text{IAR + ALR}}}}^{2}$	0.26	0.73	0.30	0.75	0.29	0.26	0.29	0.64	0.51	0.73	0.09	0.31	0.25
$R_{{{\text{IAR}} \to {\text{IAR}} + {\text{ALR}}}}^{2}$	0.16	0.05	0.04	0.14	0.12	0.01	0.24	0.44	0.25	0.52	0.04	0.08	0.00

The following statistical comparisons are reported: CHK → ADK, CHK → IAR, CHK → IAR + ALR, ADK → IAR, ADK → IAR + ALR, IAR → IAR + ALR

**Fig. 3:**
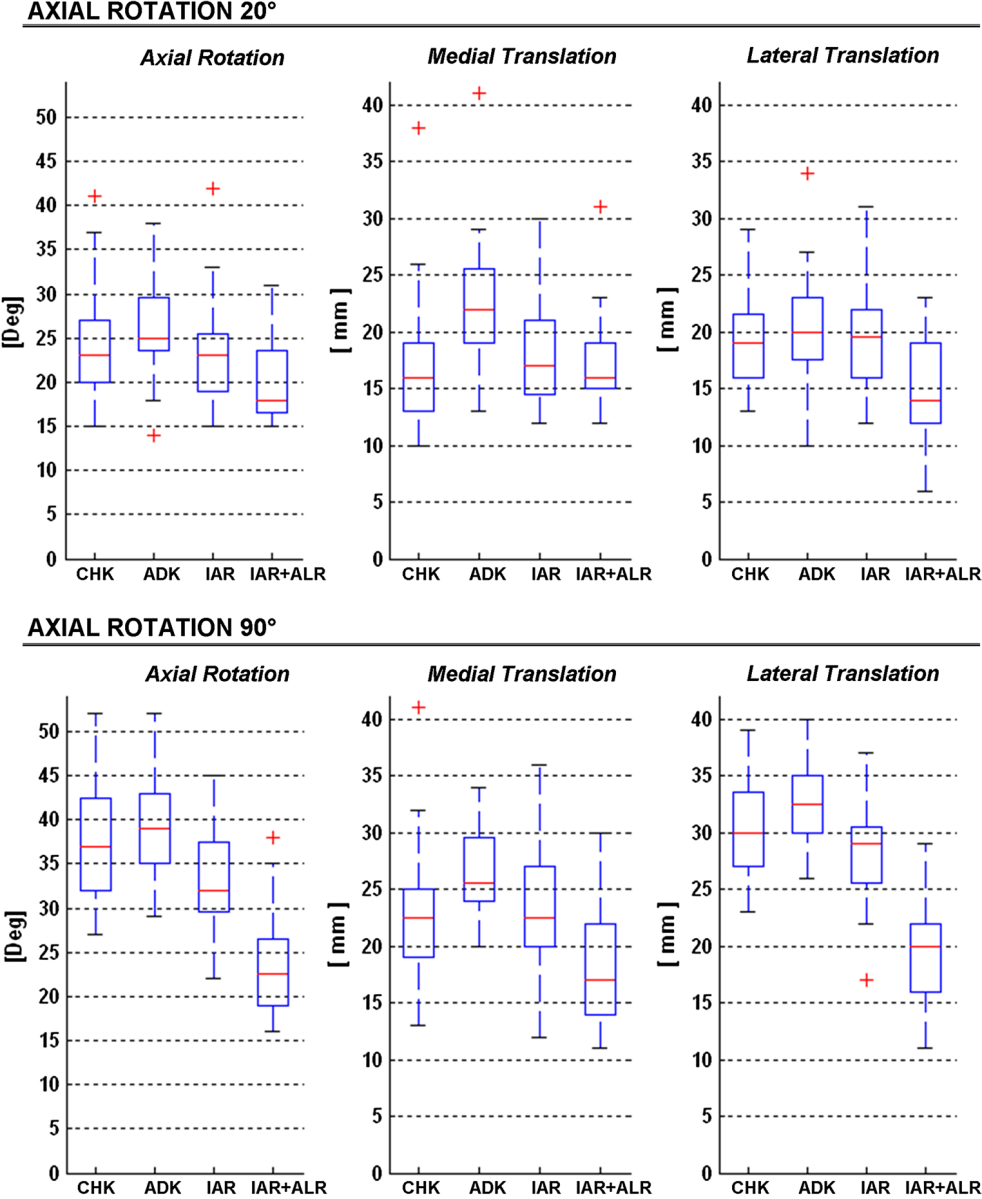
*Box plots* of axial rotation (*first column*) and the anteroposterior translations in the medial (*second column*) and lateral (*third column*) compartments, during rotational manoeuvres at 20° (*top*) and 90° (*bottom*) of knee flexion. All graphical representations match those in Fig. 2

During rotational manoeuvres, in CHK rotational laxity was larger at 90° (mean 37.5°) than that at 20° (23.9°) of knee flexion (Fig. 3; Table 2). This implied larger translations at 90° than at 20°, and also larger in the lateral than in the medial compartment (1.2 times), though the latter was less evident than in the anteroposterior drawer test (2.3 times). Axial rotation in ADK was not significantly different from that of CHK (about 10 % difference at 20° flexion and 4 % at 90°). However, anteroposterior laxity in the medial compartment was 35 % larger at 20° of knee flexion (from about 16 to 22 mm, *p* < 0.00) and 15 % larger at 90° (from about 23 to 26 mm, *p* = 0.005). Much smaller differences were found in the lateral compartment at both flexion angles. After IAR, when compared to CHK, the axial rotation was restored at 20° (from the healthy 25 to the reconstructed 24, n.s.) and over-constrained at 90° (from 37 to 34, *p* = 0.012). The translational laxity was significantly restored at the medial compartment both at 20° flexion (from about 16 to 18, n.s.) and at 90° flexion (from about 23 to 24, n.s.), as well as at the lateral compartment both at 20° flexion (about 19, n.s.) and at 90° flexion (from about 30 to 28, n.s.). After ALR, axial rotation was over-constrained both at 20° (from 24 to 20, *p* = 0.005) and at 90° (from about 37 to 24, *p* < 0.00). For the translational laxity, ALR resulted in deterioration of the laxity obtained with the IAR, except for the medial compartment at 20° (from 16 to 17, n.s.).

**Fig. 4:**
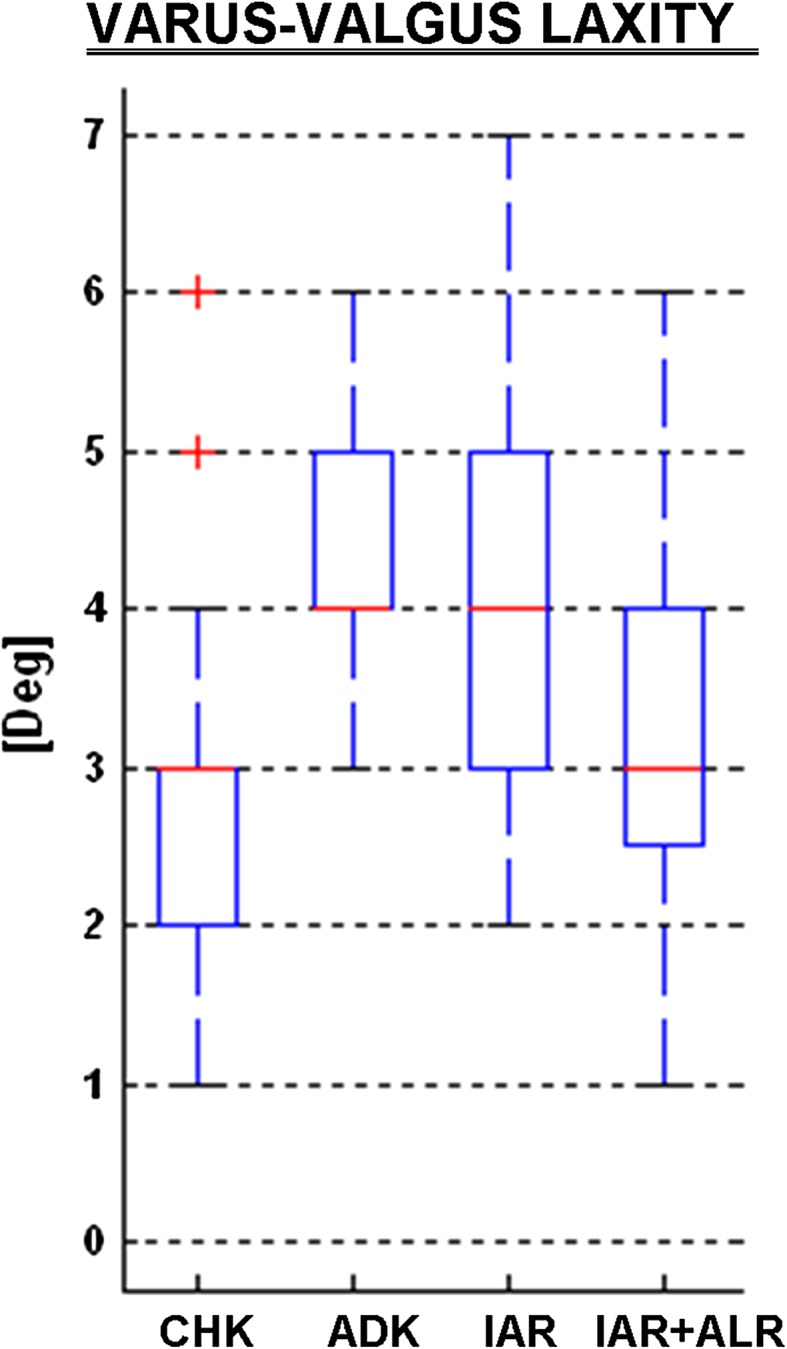
*Box plots* summarizing rotational laxity in the frontal plane (°) during varus–valgus test at 0° of knee flexion. All graphical representations match those in Fig. 2

The frontal plane rotations during the varus–valgus test (Fig. 4; Table 2) revealed a significantly larger laxity in ADK (about 4°) than that of CHK (3°), about a 47 % increment (*p* < 0.00). The physiological amounts were not restored either by the IAR (4°, *p* = 0.001) or by the additional ALR (about 4°, n.s.) technique.

**Fig. 5:**
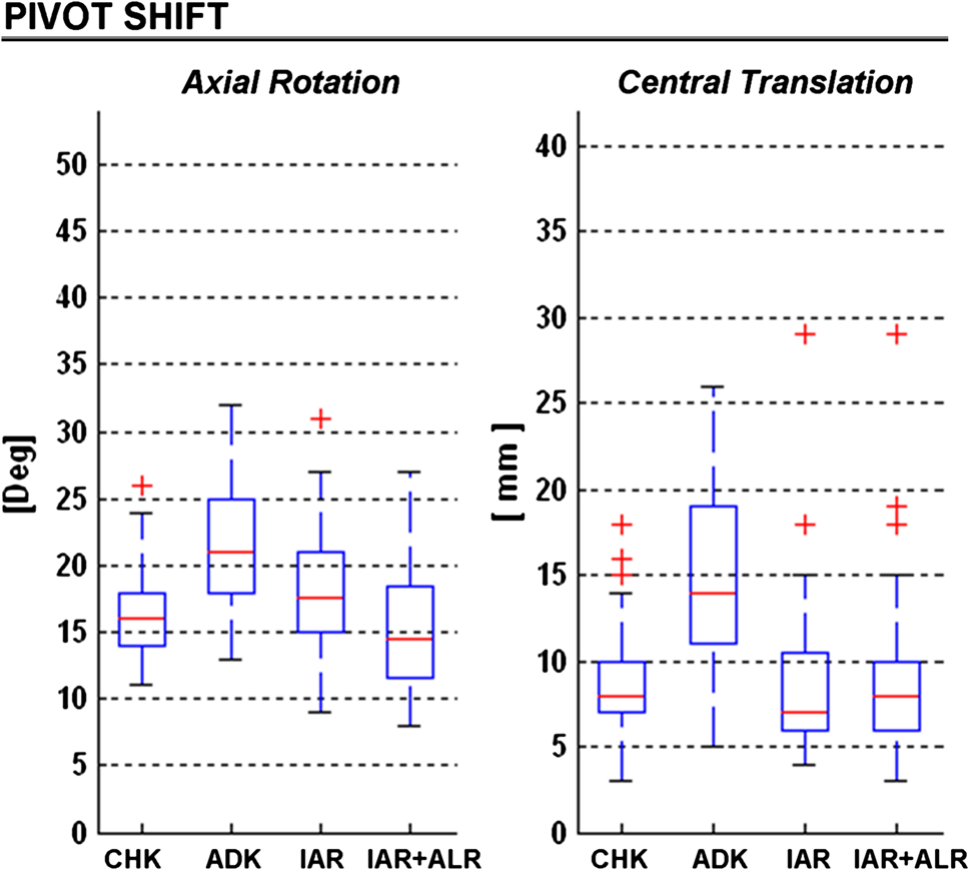
*Box plots* summarizing coupled translation and global axial rotation during pivot-shift manoeuvre. All graphical representations match those in Fig. 2

The pivot-shift manoeuvre (Fig. 5; Table 2) was the only test able to reveal significant rotational laxity in the ADK (about 21°) with respect to the CHK (about 16°, *p* < 0.00). This applied also to the translation, about 15 and 9 mm respectively (*p* < 0.00). After IAR, axial rotation nearly returned to normal values (18°, n.s.) and perhaps this was even better after ALR (15°, n.s.). Anteroposterior translation in CHK was found restored as well, by both the IAR (n.s.) and IAR + ALR (n.s.) techniques.

## Discussion

The most important finding of the present study was that intra-articular plus additional extra-articular anterolateral reinforcement procedures for ACL surgical reconstruction do not restore normal joint laxity: the isolated intra-articular reconstruction does not restore laxity in the medial compartment, and the additional reinforcement over-constrained lateral compartment. This was demonstrated in the present study where original measurements of knee laxity were taken directly in the operating theatre with bone-anchored trackers, having access also to the contralateral healthy joint. We had hypothesized that other kinematics abnormalities than the known excessive internal tibial rotation might occur after ACL rupture and persist after its intra- and extra-articular surgical reconstructions. The present results show that, in the ACL-deficient knee (the ADK), axial rotation was not significantly different except in the pivot-shift test. The anteroposterior drawer test showed a larger increase in anteroposterior laxity in the medial compartment compared with that of the lateral one. In comparison with CHK, good results were obtained with the IAR surgical technique for most of the measurements taken apart the anteroposterior laxity at medial compartment. However, additional ALR over-constrained the lateral compartment and did not restore natural anteroposterior translation in the medial compartment. In the varus–valgus test, significant residual laxity persisted; in the pivot-shift test, both surgical techniques resulted in joint laxities significantly similar to the corresponding CHK knee. It is recommended therefore to put more attention to medial extra-articular lesions and to encourage their reconstructions; in addition, extra-articular reinforcement should avoid excessive restraints to lateral compartment mobility.

The laxity measurements taken in the healthy knee (CHK) revealed itself interesting findings (Table 2). Axial rotation increased with knee flexion (Fig. 3; 57 % from 20° to 90°), as previously discussed [7]. The primary role of ACL remains that of controlling the coupled anteroposterior translation, which increased as well with knee flexion. Conversely, anteroposterior translation in the medial and lateral compartments decreased from 20° to 90° flexion (Fig. 3) because of the corresponding tensioning of the ACL, thus implying that it influences the anteroposterior drawer test. The observed larger extent of translation in the medial than lateral compartments (Fig. 3) confirms that the joint centre of rotation during flexion is located more medially [13, 27]. This difference is more pronounced during the anteroposterior drawer (2.3 times) than during rotation (1.2 times) tests, thus suggesting that this centre can move from the medial to a more lateral position in the latter test.

In the ACL-deficient knee (ADK), laxity was of course larger than that of the CHK, about two times at 20° and 1.5 times at 90° flexion in the anteroposterior drawer test, larger in the medial compartment than that of the lateral one, which supports a lateral displacement of the rotation centre [39]. This was the case also for axial rotation, though not on a statistical basis (Table 2). This lack of significant change in rotational laxity supports previous in vitro observations after isolated ACL section [29]. The significant difference with respect to CHK observed also in varus/valgus was unexpected, since pre-operative clinical examination did not reveal this frontal plane laxity in the ADK. This suggests that this laxity difference, smaller than 2° on average, can hardly be appreciated by manual testing in this standard manoeuvre.

The pivot-shift test was introduced also because it correlated with functional impairments and lower levels of activity [32]. Joint translations and axial rotations are now accepted parameters for the quantitative assessment via this test. A recent study showed good correlations also with clinical grades [21] and very similar results to those reported in the present work (Fig. 2; Table 2), i.e. one millimetre difference on average for both CHK and ADK. Only about 2° of difference in axial rotation was observed between the present and another study [34]. Measurements taken in the operating theatre via surgical navigation systems in ACL-deficient knees also match the present results for this pivot-shift test: our 15-mm mean translation compares well with that of 21 mm obtained by [50] and that of 20 mm by [5]. Our 21° of axial rotation compares well with that of 24° by [26] and that of 25° by [5]. According to [45], anteroposterior translations larger than 12 mm usually indicate concomitant injury to secondary restraints, most commonly the anterolateral capsule, lateral meniscus and ilio-tibial band.

The extent to which joint laxity after surgery is compared with that at the CHK varied by test, flexion angle and compartment (Table 2). In the anteroposterior drawer test, IAR alone restored significantly physiological laxity, in both rotation and translation at both compartments, but at 20° flexion only; at 90°, this was true only for the translation in the lateral compartment. This reduced effect in the medial compartment, although compensated after ALR, suggests that additional anatomical structures might have been damaged with injury or might have progressively deteriorated over time between injury and surgery. The associated residual varus–valgus laxity can be accounted for by possible lesions to the medial part of the knee. The posteromedial capsule must be in tension and resist valgus, posterior tibial translation and internal rotation but only with the knee extended [36, 63]. In our series of isolated ACL injuries, none of the 32 patients had a history of more than grade I medial collateral ligament lesion or an increase in varus–valgus laxity upon clinical examination. Therefore, more attention should be devoted to these anatomical structures since minor injuries at initial trauma or progressive distension before surgery can both lead to non-physiological laxity [46]. It has been suggested that peripheral anatomical structures are the primary restraints to tibial rotation [1, 43]. Peripheral anterolateral lesions were reported intra-operatively in 93 % of ACL reconstructions [55] and are commonly observed by MRI [8]. The anterolateral ligament has been described as an additional potential capsular rotational stabilizer [11]. The unsuccessful restoration of medial compartment restraint may also explain the degenerative changes which occur mostly at the medial compartment [37] even after a successful ACL reconstruction with negative pivot-shift test and normal anteroposterior translation [44]. In this respect, a minimal invasive acute medial collateral ligament stabilization has been proposed in case of partial ACL deficiency [15]. Interestingly, in our drawer test at 20°, translation in the medial compartment significantly deteriorated from IAR to ALR conditions: we hypothesize that the pivot-shift manoeuvre performed immediately after IAR resulted in a mild distension of the graft, by tensioning the implants inside the bone bed. The anterolateral reinforcement might also contribute to overloading the medial compartment by a lateral displacement of the knee rotation centre. ALR corrected significantly the varus–valgus laxity with respect to ADK, but that was not enough to return this to the physiological condition. Because it was unfeasible to separate the amount of varus and valgus laxity, it was not possible to assess whether ALR contributed to correct the latter due to lateral peripheral lesions or the former due to medial peripheral lesions. However, this test was performed in full extension, where a healthy lateral collateral ligament is tightened to prevent valgus laxity.

By looking at the pivot-shift test, IAR was able to return the knee close to physiological laxities, IAR + ALR to even a slightly better extent, although neither were significantly different from CHK. IAR was in fact expected not to reproduce fully the natural rotational control, which might be better achieved by a more “anatomic” single- or double-bundle reconstructions [22, 33]. Possible anterolateral peripheral lesions might have occurred as well in our patients at the time of injury or between injury and surgery, as documented in the literature [1, 8, 43, 55]; a relevant adjunction of an anterolateral reinforcement during ACL reconstruction has been reported [14, 40, 57]. Indeed, in our study, the IAR corrected the coupled rotation during pivot-shift test.

Laxity at the lateral compartment in all relevant tests was largely restricted. This is apparently the main drawback of the ALR, partially because this implies a lateral displacement of the rotation centre, subsequently reducing overall joint mobility, while increasing it in the medial compartment. These effects combined with a modification of the translation–rotation balance may lead to long-term degenerative changes [2, 18]. A non-isometric graft placement can be considered to address this, and though the present femoral graft insertion is the most appropriate to control tibial internal rotation [31], other authors have proposed a more posterior tibial fixation back to the posterior aspect of the Gerdy’s tubercle to enhance isometry [10, 35]. Future contribution for the best possible placement of ALR can take advantage of the recent anatomical descriptions of the anterolateral ligament [9, 11, 60]. Viscoelasticity of the graft must also be considered: the different properties with respect to natural tissue likely result in inadequate mechanical response to dynamic loading [51, 59].

The present study has several limitations. Laxity is here taken during operation and cannot reveal the following biological processes, such as tendon-to-bone healing and ligamentization affected also by patient activity, known to result in minor graft distension over time [6]. The present results were also influenced by femoral tunnel placement, here in the anatomical position via the anteromedial portal [4, 38, 53] performed consistently by the same surgeon. Results smaller than 1° and 1 mm should be considered with care because of the inaccuracies, mainly in identifying anatomical landmarks. The lack of a tourniquet and its compressive effect on the extensor apparatus might have modified the kinematic response at the healthy knee. Finally, the manual application of force and torque to the joint during the laxity tests is operator dependent, and although performed by a single surgeon, these might have been differed between patients.

## Conclusion

After the standard isolated intra-articular procedure for ACL reconstruction, good restoration of natural laxity was obtained; the additional anterolateral reinforcement resulted in over-constraint in axial rotation and at the lateral compartment, while excessive laxity still persists at the medial one. This additional procedure had a beneficial effect in the anteroposterior laxity at the medial compartment in the anteroposterior drawer test at 90° flexion, but natural laxity was not achieved at 20° flexion. Overall, with respect to the contralateral knee, ACL reconstruction by intra-articular plus additional anterolateral reinforcement does not restore normal joint laxity.
